# High birth weight as an important risk factor for infant leukemia

**DOI:** 10.1038/sj.bjc.6604202

**Published:** 2008-01-29

**Authors:** S Koifman, M S Pombo-de-Oliveira, and The Brazilian Collaborative Study Group of Infant Acute Leukemia

**Affiliations:** 1 National School of Public Health/FIOCRUZ, Rua Leopoldo Bulhões, 1480, Rio de Janeiro, RJ 21041-210, Brazil; 2 Divisão de Medicina Experimental, Centro de Pesquisa – Instituto Nacional De Câncer, Rio de Janeiro, Rua André Cavalcanti, 37, CEP, Rio de Janeiro, RJ 20231-050, Brazil

**Keywords:** infant acute leukaemia, intrauterine factors, birth weight, *MLL* rearrangements

## Abstract

In this paper, we compared the birth weight distribution among 201 infant leukaemia (IL) cases with that of 440 noncancer controls enrolled in Brazil in 1999–2005. Compared with the general population and the stratum 2500–2999 g as reference, IL cases weighing 3000–3999 g presented an odds ratio (OR) of 1.68 (95% CI: 1.03–2.76), and those of 4000 g or more, an OR of 2.28 (95% CI: 1.08–4.75), *P*_trend_<0.01. Using hospital-based controls, the OR for 4000 g or more, compared to 2500–2999 g, was 1.30 (95% CI: 1.02–1.43) after adjusting for confounders (gender, income, maternal age, pesticide and hormonal exposure during pregnancy). The results suggest that high birth weight is associated with increased risk of IL.

The positive association between birth weight and increased risk of childhood leukaemia has been reported in industrialised countries (Westergaard *et al*, 1997; Hjalgrim *et al*, 2003). Acute lymphoblastic leukaemia (ALL) or acute myeloblastic leukaemia (AML) affecting infants (under 12 months), which have been little studied, often contain a chromosome break following the recombination of the *MLL* gene with other genes (Felix and Lange, 1999). It has been hypothesised that malignant clones, which will promote acute leukaemias in early infancy would result from maternal exposures to such environmental agents as dietary items and chemicals inhibitors of DNA topoisomerase II activity (Ross *et al*, 1996b; Alexander *et al*, 2001).

In a recent molecular case–control study in Brazil of infant leukaemia (IL) with *MLL* gene rearrangements, a strong and significant association between maternal use of hormones during pregnancy and IL was observed (Pombo-de-Oliveira *et al*, 2006). Also of relevance, oestriol, progesterone and prolactin measured during pregnancy are reported as positively associated with birth weight and cancer risk in offspring (Mucci *et al*, 2003; Nagata *et al*, 2006). We have therefore examined IL risk in relation to birth weight using an existing database constructed by the Brazilian Collaborative Study Group of Infant Acute Leukaemia (BCSGIAL).

## MATERIALS AND METHODS

The BCSGIAL is a cooperative study group in different states of Brazil running a hospital-based case–control study to evaluate associations with IL maternal exposures to environmental risk factors during pregnancy. Details regarding case definition, control selection, exclusion criteria and data collection have been published previously (Emerenciano *et al*, 2006; Pombo-de-Oliveira *et al*, 2006).

In this report, IL cases were included whenever birth weight data were available. Controls were children, matched for age strata and gender, with life-threatening conditions in the same hospitals as the cases, excluding malignancy. Data on birth weight were obtained from the health-care cards, regulated by the Brazilian National Health System.

Birth weight data from IL cases were either compared with hospital-based controls, or with population-based data relating to all births in the general population of Brazil in the year 2000, and consolidated by the Brazilian National Health System (Ministério da Saúde, 2007). We ascertained birth weight by gender, leukaemia type (ALL and AML) and *MLL* status. Crude and adjusted odds ratios (ORs), and their 95% confidence intervals, were ascertained for the birth weight strata (2500 g or less; 2500–2999 g; 3000–3499 g; 3500–3999 g; and 4000 g or more) adjusted for selected confounders (gender, income, maternal age, hormonal intake and pesticide exposure during pregnancy). The available national data on birth weight merged 3000–3499 g and 3500–3999 g strata, and this was followed with IL birth weight to allow the relevant comparisons. Statistical analyses were performed using unconditional logistic regression models, as described elsewhere (Pombo-de-Oliveira *et al*, 2006) and using the packages SSPS, version 13.5 (SPSS, Chicago, IL, USA). All collaborating institutions approved the BCSGIAL Study, and written consent was obtained for diagnostics procedures and for interviews with the mothers.

## RESULTS

A total of 641 subjects, 201 cases (148 ALL and 53 AML) and 440 controls, stratified by age at diagnosis (0–12 *versus* 13–21 months), were enrolled in 1999–2005. The numbers of the main clinical and laboratory data were same as described previously (Emerenciano *et al*, 2006), but eight AML cases were excluded due to lack of birth weight records. Eight cases, which were misclassified in the previous report as biphenotypic leukaemia with aberrant phenotype, were then categorized as ALL in the present analysis.

Low birth weight (<2500 g) was found in 16 (8.0%) cases and in 74 (16.8%) controls. The comparison between IL and controls birth weight distributions showed that both ALL and AML weighed on average 3488 g at birth, compared with 3226 g among controls (*P*=0.0002); median birth weights were 3310 and 3226 g for ALL and AML, respectively. Children with ALL had slightly higher birth weights than AML, while male infants were slightly heavier than female infants. The birth weight for male IL infants was 3349.2 g (s.d.=49.6), with 95% CI: 3250.9–3447.5; for female IL infants, the birth weight was 3262.8 g (s.d.=48.8), 95% CI: 3166.0–3359.7. Further stratification by age revealed minimal differences between the strata before or after 12 months of age at diagnosis of IL (data not shown). It should, however, be remarked that *MLL* status in the current study was unknown for a larger proportion of children with low birth weight.

Data were stratified according to *MLL* gene status (positive, negative and unknown). Mean birth weight in *MLL*^+ve^ cases (*n*=68) was 3340 g (s.d.=64.85), while in *MLL*^−ve^ cases (*n*=80), it was 3310 g (s.d.=51.2); the medians were 3310 and 3300 g, respectively.

The magnitude of the association with birth weight IL was also explored in increasing weight strata (Tables 1 and 2). Compared to birth weights 2500–2999 g, higher weights showed increased ORs for either ALL or AML regardless of the control, population or being hospital-based. Compared with the general population, ALL cases weighing 3000–3999 g showed an OR of 1.68 (95% CI: 1.03–2.76), and those weighing 4000 g or more, an OR of 2.28(95% CI: 1.08–4.75, *P*<0.01, Table 1). The cases that were *MLL*^+ve^ showed a higher birth weight than *MLL*^−ve^ cases at ages 12–21 months, and an inverse relationship in the first year of life was observed (figures available online).

## DISCUSSION

Despite the many studies on childhood leukaemia and birth weight, few have focused on IL exclusively (Ross *et al*, 1997; Yeazel *et al*, 1997), which is in spite of the value of risk factors specifying the age group and the childhood leukaemia subtypes (Ma *et al*, 2005). The main strength of our study is probably the almost complete ascertainment of IL in all Brazil except the North region, Amazon.

In a population-based cohort, a positive linear relation was reported between birth weight, and childhood ALL and AML (Paltiel *et al*, 2004; Mclaughlin *et al*, 2006), more marked in AML among infants (hazard ratio=8.14, 95% CI: 1.8–38.9 at age 0–1 years), being particularly strong among female infants (*P*=0.001) (Paltiel *et al*, 2004). Among the variables included in this study, including maternal origin, socioeconomic status, birth weight of sibling higher than 3500 g and family size, only birth weight retained borderline significance (Paltiel *et al*, 2004). This association has been explored in the present study using different approaches, for IL ALL, AML and *MLL* status: according to a single weight cut-point, and also exploring the magnitude of association among subsequent birth weight strata. All approaches yielded results that suggested an association between high birth weight and IL.

**Figure 1:**
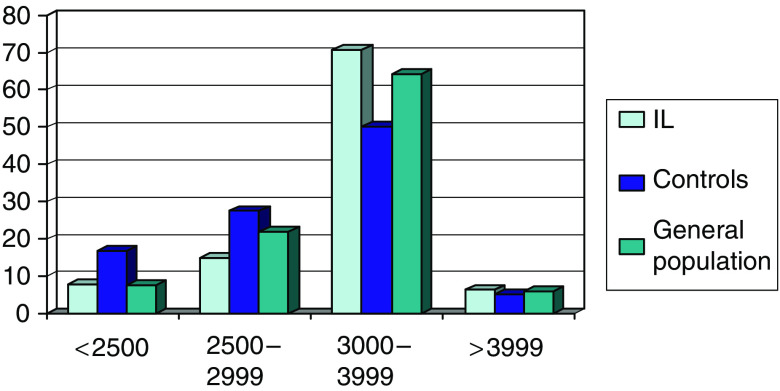
Birth weights (in g) strata distribution, IL cases, controls and the general population, Brazil.

In our study, low birth weight was more common among controls (16.8%) than IL cases (8.0%), which may have estimates of the magnitude of the association with birth weight (Figure 1). Comparisons with the general population were therefore important to control for bias in our hospital-based controls. In this connection, three different OR estimates with birth weight were obtained with the general population, providing unbiased estimates but without adjusting confounders, crude ORs using hospital controls and adjusted ORs using hospital controls.

Comparisons with the general population revealed an insignificantly increased trend in ORs between ALL and birth weight (Table 1) compared with children weighing 2500–2999 g at birth. Those weighing 3000–3999 g showed an OR of 1.69 (95% CI: 1.03–2.76), and those born with 4000 g or more, an OR of 2.28 (95% CI: 1.08–4.75), suggesting a dose–response effect (*P*<0.01). A quite similar trend with a borderline statistical significance was also observed for *MLL*^+ve^ cases (Table 1; *P*=0.059).

Birth weight strata of hospital controls suggested a dose–response effect for IL, and also for ALL and AML subtypes after adjusting for certain confounders. For AML, discordant results have been reported with birth weight (Ross *et al*, 1997; Westergaard *et al*, 1997; Yeazel *et al*, 1997; Hjalgrim *et al*, 2003). Despite no AML infants weighed more than 4000 g, suggestive dose–response trend was indeed observed.

With respect to the biological mechanisms underlying birth weight and IL development, one of the factor is hormone intake during pregnancy to induce abortion to the index pregnancy (Ou *et al*, 2002). In a previous report, we observed a high association between hormone intake during pregnancy and IL with an OR of 8.76, 95% CI: 2.85–26.93 (Pombo-de-Oliveira *et al*, 2006). Women used oral contraceptive pills in the belief that they caused miscarriage, because abortion is illegal in Brazil. Although it could be hypothesised that the association with birth weight was partly due to such hormonal intake during the pregnancy, inducing an increase in birth weight.

Although our results seem to point to an independent effect of birth weight, it has been suggested that rather than birth weight *per se*, the accelerated growth during pregnancy is the main process leading to IL (Milne *et al*, 2007). On the other hand, the United Kingdom Childhood Cancer Study has reported that babies who developed leukaemia were heavier at birth (>4000 g, OR of 1.2, 95% CI: 1.0–1.4), as were their older siblings (>4000 g, OR of 1.4, 95% CI: 1.0–1.9) (Roman *et al*, 2005). Other mechanisms suggested for the association with birth weight include the supposition that IL is initiated by aberrant gene fusions, mainly with *MLL* rearrangements (Ross *et al*, 1996a; Alexander *et al*, 2001). Interactions between high birth weight, prenatal oestrogen exposure and leukaemia risks have also been suggested (Ross *et al*, 1996a; Baik *et al*, 2005; Ross, 2006).

An association has recently been reported between ALL and birth weight among *MLL*^+ve^ cases, but not among *MLL*^−ve^ ones (Spector *et al*, 2007). In our study, a similar pattern was observed, with a borderline linear trend suggestive of a causal relationship.

It has been suggested that insulin-like growth factor-1 (IGF-1) promotes a proliferative advantage to damaged cells (Ross *et al*, 1996a; Ross, 2006). The link between birth weight, steroid hormones in intrauterine life such as IGF-1 and cancer risk indicates that such hormones tend to increase the number of stem cells and, by extension, more proliferating immature cells are exposed to harmful events, and consequently become more susceptible to malignant transformation (Cavalieri *et al*, 1997; Boyne *et al*, 2003). Whether enhanced cell proliferation and genotoxic metabolites act jointly in an additive or synergistic fashion, resulting in expansion of clonal cells with gene fusion rearrangements, and clinical IL in high birth weight babies, warrants further investigation in experimental models.

## Figures and Tables

**Table 1:** Birth weight distribution, ALL, AML, *MLL* status (1999–2005) and all births in Brazil (general population), 2000

**Birth weight**	**General population**	**IL**		**ALL**		**AML**		***MLL* ^+ve^**		***MLL* ^−ve^**	
**(in g)**	**(all births)**	***n***	**OR (95% CI)**	***n***	**OR (95% CI)**	***n***	**OR (95% CI)**	***n***	**OR (95% CI)**	***n***	**OR (95% CI)**
<2500	243 835	16	1.52 (0.80–2.89)	11	1.50 (0.72–3.11)	5	1.59 (0.46–5.16)	6	1.56 (0.51–4.54)	3	0.61 (0.14–2.26)
2500–2999	696 925	30	1.00	21	1.00	9	1.00	11	100	14	1.00
3000–3999	2 036 925	143	1.63 (1.08–2.47)	103	1.68 (1.03–2.76)	39	1.48 (0.64–3.28)	43	1.34 (0.67–2.75)	58	1.42 (0.77–2.66)
>3999	189 476	13	1.59 (0.79–3.17)^a^	13	2.28 (1.08–4.75)^b^	0	—	8	2.68 (0.99–7.15)^c^	5	1.31 (0.41–3.89)^d^

ALL=acute lymphoblastic leukaemia; AML=acute myeloblastic leukaemia; CI=confidence interval; IL=infant leukaemia; OR=odds ratio.

a *χ*^2^ for trend=4.60, *P*=0.03.

b *χ*^2^ for trend=6.66, *P*<0.01.

c *χ*^2^ for trend=3.56, *P*=0.059.

d *χ*^2^ for trend=0.91, *P*=0.34.

**Table 2:** Birth weight distribution by weight strata, IL and hospital-based controls, Brazil, 1999–2005

		**All IL**			**ALL**			**AML**		
**Birth weight**	**Hospital-based**		**Crude**	**Adjusted^a^**		**Crude**	**Adjusted^a^**		**Crude**	**Adjusted^a^**
**(in g)**	**controls**	***n***	**OR (95% CI)**	**OR (95% CI)**	***n***	**OR (95% CI)**	**OR (95% CI)**	***n***	**OR (95% CI)**	**OR (95% CI)**
<2500	74	16	0.87 (0.42–1.79)	0.88 (0.41–1.86)	11	0.86 (0.36–1.99)	0.99 (0.41–2.42)	5	0.91 (0.25–3.12)	0.74 (0.21–2.55)
2500–2999	121	30	1.00	1.00	21	1.00	1.00	9	1.00	1.00
3000–3499	150	81	2.18 (1.31–3.63)	1.23 (1.04–1.46)	57	2.19 (1.18–2.93)	1.24 (1.02–1.50)	24	2.15 (0.91–5.20)	1.20 (0.91–1.58)
3500–3999	72	62	3.47 (1.00–6.08)	1.29 (1.12–1.48)	46	3.68 (1.96–6.96)	1.30 (1.11–1.52)	15	2.80 (1.08–7.36)	1.23 (0.98–1.55)^b^
>3999	23	13	2.28 (0.96–5.37)	1.20 (1.02–1.43)^c^	13	3.26 (1.32–8.01)	1.31 (1.09–1.57)^d^	—		

ALL=acute lymphoblastic leukaemia; AML=acute myeloblastic leukaemia; CI=confidence interval; IL=infant leukaemia; OR=odds ratio.

a Adjusted for sex, income, maternal age, pesticide exposure and hormonal intake during pregnancy.

b *χ*^2^ for trend=5.51, *P*=0.018.

c *χ*^2^ for trend=16.70, *P*=0.00004.

d *χ*^2^ for trend=18.14, *P*=0.00002.

## APPENDIX

***Brazilian Collaborative Study Group of Infant Acute Leukemia***

Paulo Ivo C Araújo^1^, Jozina Aquino^2^, Jose Andréa Yunes^3^, Reinaldo Del Belo^4^, Ricardo Bigni^4^, Lilian Burlemaqui^2^, Tereza Cristina Cardoso, Maurício Dumond^2^, Fernando Augusto de Freitas^3^, Eni Guimarães Carvalho^5^, Virginia M Coser^6^, Maria Célia Moraes Guerra^1^, Maria Lucia Lee, Núbia Mendonça^2^, Isis Q Magalhães^7^, Flávia Pimenta^8^, Gilberto Ramos^9^, Terezinha JM Salles^10^, Ednalva Leite^10^, Carmen M Mendonça^11^, Flávia Nogueira^2^ and Fernando Werneck^12^

^1^Instituto de Pediátria e Puericultura Martagão Gesteira, UFRJ, Rio de Janeiro;

^2^Sociedade de Oncologia da Bahia, Salvador-Bahia;

^3^Centro Infantil de Investigações Hematológicas D Boldrini, Campinas, São Paulo;

^4^Centro de Pesquisa and Serviço de Hematologia, Instituto Nacional de Câncer, Rio de Janeiro;

^5^Hospital Martagão Gesteira, Salvador-Bahia;

^6^Departamento de Hematologia, Universidade de Santa Maria, Rio Grande do Sul;

^7^Hospital de Apoio Brasília, Unidade de Onco-Hematologia Pediátrica, Brasília, DF;

^8^Hospital Napoleão Laureano, João Pessoa, Paraiba;

^9^Departamento de Pediatria, Faculdade de Medicina, UFMG, Belo Horizonte, MG;

^10^Hospital Oswaldo Cruz, CEON, Recife, Pernambuco;

^11^Serviço de Oncologia do Hospital Joana de Gusmão Florianópolis, Santa Catarina;

^12^Departamento de Pediatria, Hospital dos Servidores do Estado do Rio de Janeiro, Rio de Janeiro
